# Benefits of symbiotic ectomycorrhizal fungi to plant water relations depend on plant genotype in pinyon pine

**DOI:** 10.1038/s41598-023-41191-5

**Published:** 2023-09-02

**Authors:** Sanna Sevanto, Catherine A. Gehring, Max G. Ryan, Adair Patterson, Adrian S. Losko, Sven C. Vogel, Kelsey R. Carter, L. Turin Dickman, Michelle A. Espy, Cheryl R. Kuske

**Affiliations:** 1 Earth and Environmental Sciences Division, Los Alamos National Laboratory, MS J495, PO Box 1663, Los Alamos, NM 87545 USA; 2 Department of Biological Sciences and Center for Adaptable Western Landscapes, Northern Arizona University, Flagstaff, AZ 86011 USA; 3 Material Sciences and Technology Division, Los Alamos National Laboratory, Los Alamos, NM 87545 USA; 4 Forschungs-Neutronenquelle Heinz Maier-Leibnitz, 85748 Garching, Germany; 5 Engineering Technology and Design Division, Los Alamos National Laboratory, Los Alamos, NM 87545 USA; 6 Biosciences Division, Los Alamos National Laboratory, Los Alamos, NM 87545 USA; 7 Present Address: Integral Ecology Group, Duncan, BC V9L 6H1 Canada

**Keywords:** Microbiology, Plant sciences, Ecology

## Abstract

Rhizosphere microbes, such as root-associated fungi, can improve plant access to soil resources, affecting plant health, productivity, and stress tolerance. While mycorrhizal associations are ubiquitous, plant–microbe interactions can be species specific. Here we show that the specificity of the effects of microbial symbionts on plant function can go beyond species level: colonization of roots by ectomycorrhizal fungi (EMF) of the genus *Geopora* has opposite effects on water uptake, and stomatal control of desiccation in drought tolerant and intolerant genotypes of pinyon pine (*Pinus edulis* Engelm.). These results demonstrate, for the first time, that microorganisms can have significant and opposite effects on important plant functional traits like stomatal control of desiccation that are associated with differential mortality and growth in nature. They also highlight that appropriate pairing of plant genotypes and microbial associates will be important for mitigating climate change impacts on vegetation.

## Introduction

In recent years, the potential of rhizosphere microbes to improve plant performance has been recognized. Numerous attempts at harnessing microbes to increase plant productivity and stress tolerance have been reported both in agricultural and forestry settings^1^. With climate predictions showing increased frequency and severity of drought in many areas around the world^2^, understanding how microbial symbionts affect, and can improve, plant drought tolerance is paramount for maintaining vegetation and the ecosystem benefits it provides^3^.

Ectomycorrhizal fungi (EMF) colonize the roots of woody perennials where they enhance soil resource uptake in exchange for fixed carbon from their hosts^4,5^. Through physical and biochemical interactions, EMF can influence plant traits and environmental responses^6^, and improve the ability of their hosts to resist, tolerate, and recover from drought^7–9^. EMF, for example, can alter the anatomy and hydrophilic properties of roots, influencing the apoplastic water uptake pathway^10^. They can also form an extensive network of hyphae in the soil that concentrates water supply toward host roots, particularly in fungal species that form cord-like strands called rhizomorphs^10^. While colonization by EMF can reduce root biomass and length as the root tips thicken and are enveloped by a mantle of fungal hyphae^5^, the enhanced access to water can increase stomatal conductance^7,11^ and potentially allow greater carbon assimilation and survival under drought conditions.

In addition to these direct impacts on plant function, EMF may also influence fundamental plant traits that determine plant drought sensitivity. One such trait, stomatal closure point (SCP), or the leaf water potential at which plants close stomata to avoid desiccation, has become an important metric for estimating plant growth capacity and survival under drought^12–15^. In determining the drought severity where a plant turns from a carbon sink to a carbon source, SCP is an integrated measure of both plant desiccation tolerance, and carbon assimilation capacity under drought with consequences both at the individual plant and ecosystem scales^16,17^. Across the plant kingdom, SCPs fall along a continuum^18^. Within species, SCP is thought to be relatively constant because it is related to plant hydraulic anatomy^19^, although, there is some evidence that growth stage and environment can influence SCP^20–22^. Specifically, SCP has been related to xylem vulnerability to loss of hydraulic conductivity via embolism^19^, which is linked to xylem anatomy, water transport capacity^23^, plant productivity, and growth rates^24,25^. Species in areas with abundant access to water and nutrients grow faster, have higher leaf area and wider xylem conduits. These plants are more vulnerable to embolism than plants growing under limited resources and manifest a higher SCP to protect the vulnerable hydraulic pathways^24,26–28^. While less efficient in water transport, plants with lower SCP (desiccation tolerating plants) are more productive under drought and recover faster^13,29^.

In the semi-arid Southwest USA, two genotypes of pinyon pine (*Pinus edulis* Engelm) that have different resistance to drought mortality and show different growth under drought conditions co-occur^30–32^. These pinyon genotypes are part of the pinyon-juniper woodlands that have become a model system for studies to understand the links between plant hydraulics, carbon uptake, SCP and plant mortality. The co-existing pinyon and juniper species have different SCPs under drought leading to theories of drought induced hydraulic failure and carbon starvation as mechanisms of plant mortality^12,16,33,34^. Because the drought tolerant and intolerant pinyon genotypes are of the same species, they are expected to be similar in anatomy, but they consistently differ in their root-associated microbial composition in both natural and common garden conditions^31,32,35^, indicating that drought tolerance and microbial community composition have a genetic basis. The mechanisms to explain the differences in growth and survival under drought, however, are unknown.

To shed light on the impacts of symbiotic EMF on plant water relations, and the mechanisms leading to improved drought survival of the drought tolerant genotype, we conducted an experiment on seedlings of the drought tolerant and intolerant pinyon variants. Seedlings of both genotypes were grown from seeds inoculated with live and sterilized rhizosphere microbiome of drought tolerant maternal trees, dominated by EMF of the genus *Geopora*. Based on previous knowledge of differences in growth, and EMF preferences of these genotypes^31,32,35^, as well as observed similarities of the effects of EMF on pine function at family level^36^ and impacts of improved water uptake on plant growth^37^, we hypothesized that (1) colonization by EMF in the genus *Geopora* will increase water transport and uptake in both genotypes, but the effect will be larger in drought tolerant than intolerant seedlings, (2) the improved water transport and uptake will result in larger seedling growth in live than sterile inoculated plants of both genotypes, and consequently, (3) greater EMF colonization will result in higher SCP through increased water availability and resultant effects on plant growth and hydraulic vulnerability. To test our hypotheses, we used neutron radiography to track and compare in situ root and rhizospheric soil water uptake of plants treated with live or sterile inoculum. This technique uses a particle accelerator to image the movement of heavy water (D_2_O) inside the plant or soil in real time.We also measured transpiration, root colonization by EMF, above- and belowground drought-tolerance-related plant traits including SCPs.

## Results

**Figure 1:**
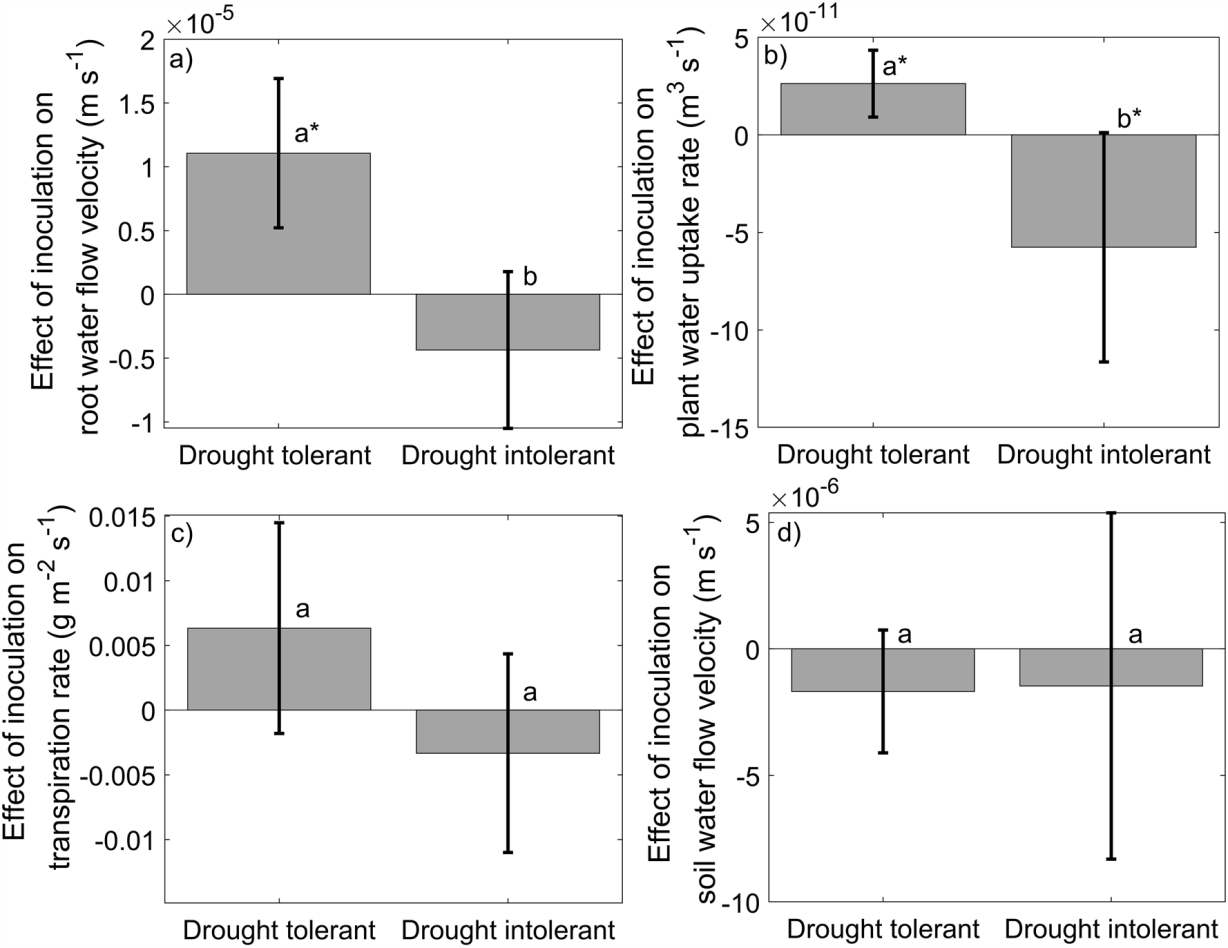
Effect of the inoculation treatment on root water flow rate (**a**), plant water uptake rate (**b**), plant transpiration rate (**c**) and soil water flow rate (**d**) in the drought tolerant and drought intolerant plant genotypes. The effect is calculated as the difference between the live and sterile inoculated plants. Positive values indicate an increase resulting from live inoculation compared to sterile inoculated controls. The error bars show standard deviation. Statistically significant differences between plant genotypes are marked by different letters. Changes that are statistically significantly different from zero are marked with an asterisk.

Inoculation with live EMF increased root water flow velocity and uptake in the drought tolerant genotype, but it decreased both water flow velocity and uptake in the drought intolerant seedlings (F = 11.23 and F = 5.56 respectively, p < 0.05; Fig. 1a, b). The inoculation had minimal effects on transpiration rates (F = 1.46 p = 0.35, Fig. 1c) although the tendency was similar as the effect on water flow rates for both genotypes. Inoculation with EMF had no effect on soil water flow velocity (F = 0.0, p = 0.96) (Fig. 1d).

**Figure 2:**
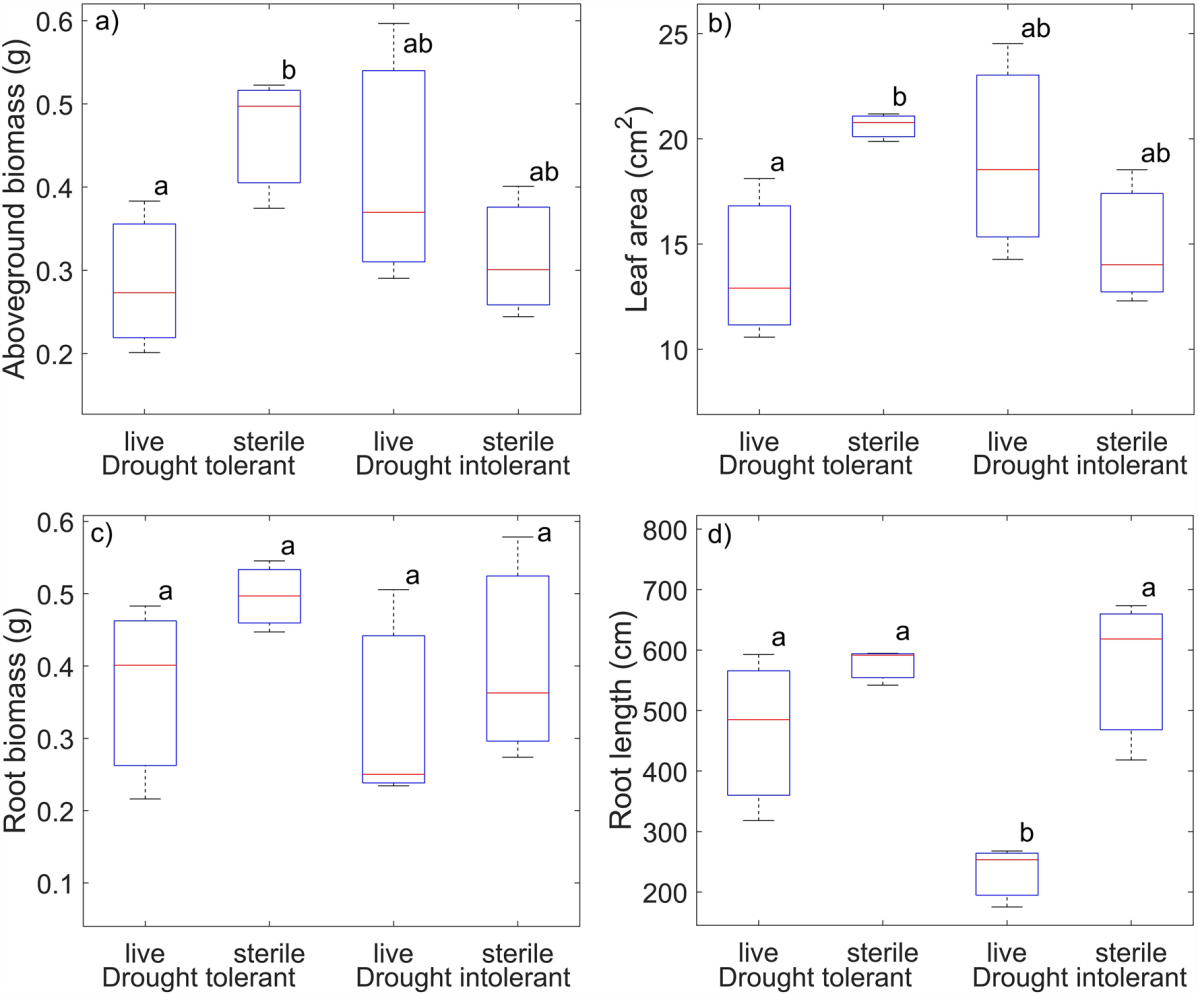
Effect of live and sterile inoculation treatments on aboveground biomass (**a**), leaf area (**b**), root biomass (**c**), and root length (**d**) in drought tolerant and intolerant pinyon pine seedlings. The error bars show standard deviation. Statistically significant differences between inoculation treatments and plant genotypes are marked by different letters.

The inoculation treatment influenced plant growth more in the drought tolerant than in the drought intolerant seedlings (Fig. 2). However, the live inoculation treatment reduced, rather than increased, aboveground biomass (F = 6.54, p = 0.06) and leaf area (F = 8.9, p = 0.04). In contrast to the effect on drought tolerant seedlings, live inoculation had no effects on the aboveground biomass or leaf area in drought intolerant seedlings, despite a consistent trend of increased biomass in live inoculated plants (Fig. 2a, b). Generally, aboveground biomass and leaf area were largest in the sterile and smallest in live inoculated drought tolerant seedlings, with drought intolerant seedlings between these extremes. In contrast to aboveground growth, belowground growth was influenced more by inoculation in the drought intolerant than drought tolerant seedling (Fig. 2c, d). Inoculation treatment had no effects on root biomass in either treatment (F = 0.8, p = 0.4), but live inoculation reduced root length in the drought intolerant genotype (F = 27.42, p < 0.001) (Fig. 2d). The difference in root length was not reflected in the total root biomass (Fig. 2c), suggesting that the root length decrease was due to decrease in very fine root length, consistent with root tip thickening and EMF hyphal envelopment^5^.

**Figure 3:**
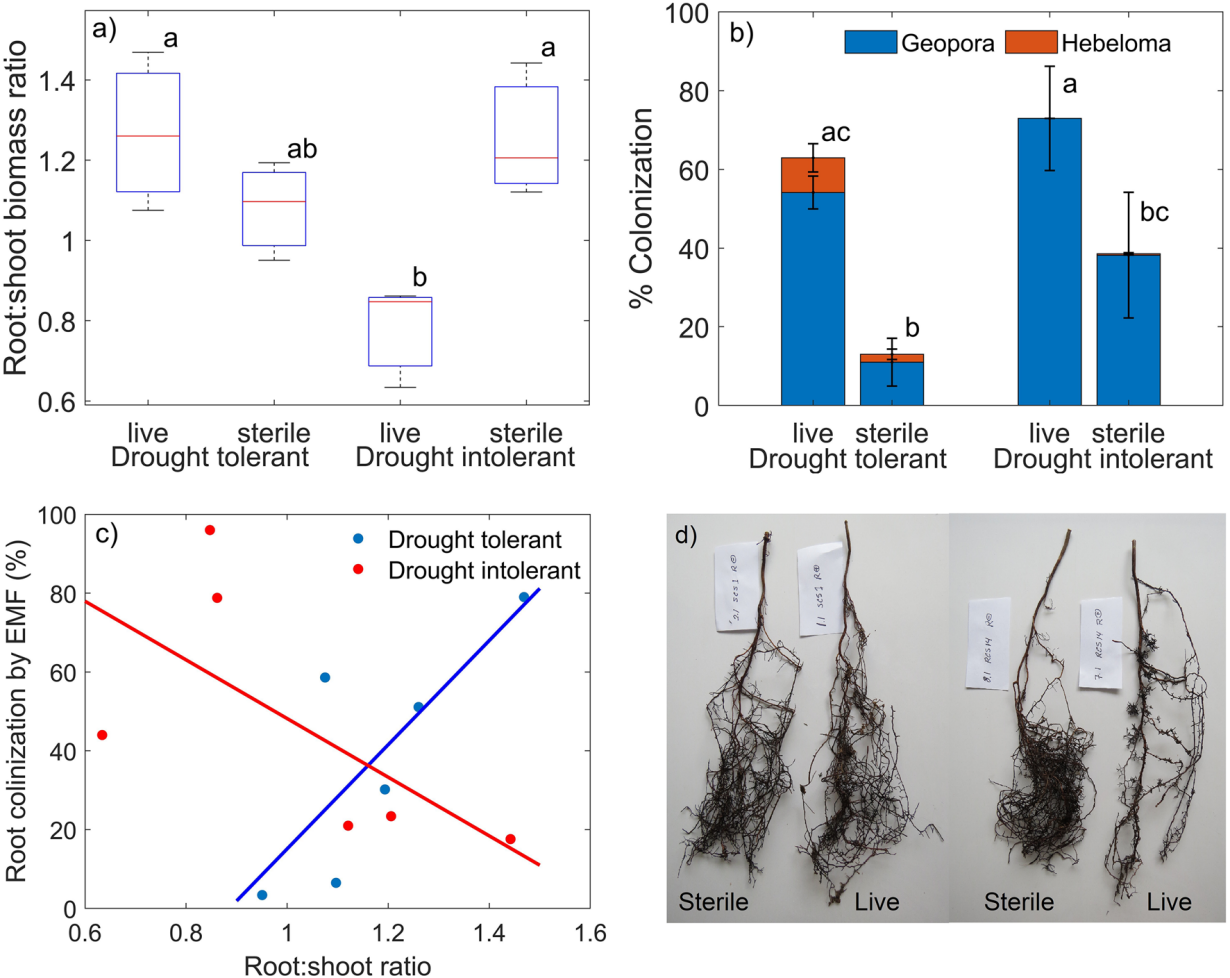
Effect of live and sterile inoculation treatments on root:shoot ratio (**a**), root colonization by EMF (**b**) in drought tolerant and intolerant pinyon pine seedlings. The error bars show standard deviation. Statistically significant differences between inoculation treatments and plant genotypes are marked by different letters. (**c**) The relationship of root EMF colonization with root:shoot ratio in drought tolerant (blue; c = 0.79, R^2^ = 0.62 p < 0.01) and intolerant (red; c = −0.65, R^2^ = 0.43, p < 0.05) genotypes. (**d**) Examples of root systems of sterile and live inoculated drought tolerant (left) and drought intolerant (right) seedlings.

Overall, the live inoculation led to reduced root:shoot ratio in drought intolerant plants (F = 13.49, p < 0.01), while the inoculation treatment had no effect on drought tolerant plant root:shoot ratio (Fig. 3a). Inoculation with live EMF resulted in higher root colonization with EMF than treatment with sterile inoculum in both plant genotypes (F = 27.42, p < 0.001 drought tolerant; F = 11.54, p = 0.03 drought intolerant) (Fig. 3b). Average EMF colonization was similar in the live inoculated treatments of drought tolerant and intolerant seedlings, but the colonization was slightly higher in the sterile inoculated drought intolerant than tolerant seedlings. The EMF consisted mostly of *Geopora pinyonensis*, which accounted for > 92% of all EMF*. Geopora pinyonensis* is the most common EMF associate of the drought tolerant *P. edulis* where the inoculum was collected^31,35^, demonstrating successful inoculation. The only other EMF species present was a member of the genus *Hebeloma*. Species of this genus were more common in the drought tolerant than intolerant seedlings (F = 5.61, p < 0.05), but always represented only a small proportion of the colonized root tips. The amount of EMF colonization correlated with the root:shoot ratio in both plant genotypes, but the correlations were in opposite directions (Fig. 3c). In the drought tolerant seedlings, root:shoot ratio increased with increasing colonization (c = 0.79, R^2^ = 0.62 p < 0.01), while in the drought intolerant seedlings root:shoot ratio decreased with increasing colonization (c = −0.65, R^2^ = 0.43, p < 0.05). These differences emerged even though we selected plants of similar aboveground size for water flow/uptake measurements and were driven by the opposing effects of live inoculation on the root systems of drought tolerant and intolerant plants (Figs. 2 and 3d).

**Figure 4:**
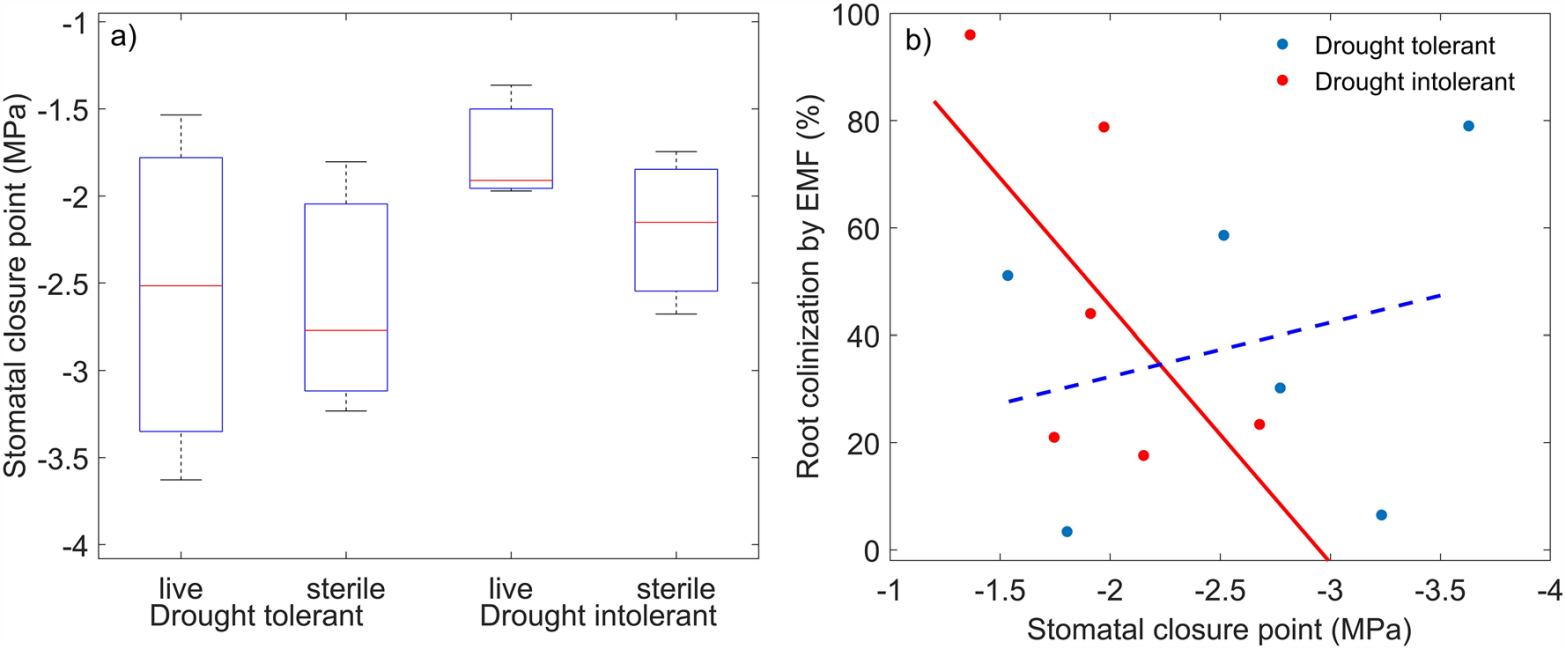
The effects of live and sterile inoculation on stomatal closure point (SCP) in drought tolerant and intolerant pinyon pine seedlings (**a**), and the correlation of SCP with root EMF colonization in drought tolerant (blue) and drought intolerant (red) seedlings (**b**). There were no statistically significant differences in SCP between the treatments or plant genotypes, but SCP correlated positively with root EMF colonization in the drought intolerant genotype, with stomatal closure point increasing at the rate of 1 MPa/48% increase in colonization (R^2^ = 0.4, p < 0.05).

Even though the seeds for this study were collected from maternal sources known to differ in growth and survival under severe drought, we did not observe differences in SCP between the plant genotypes or inoculation treatments (Fig. 4a). The average SCP of the drought tolerant seedlings was 0.5 MPa lower than that of the drought intolerant seedlings, but the large variation in SCPs of the drought tolerant plants dampened our ability to detect differences between seed sources. In drought intolerant seedlings, SCP correlated with root EMF colonization (c = −0.63, p < 0.05), such that SCP increased with EMF colonization at roughly 1 MPa per 48% increase in colonization (Fig. 4b). For these plants, EMF colonization explained 40% (R^2^ = 0.40) of the variation in SCP. In drought tolerant seedlings, SCP tended to decrease with increasing colonization, but there was no correlation (Fig. 4b).

## Discussion

Our results show that root colonization by the same EMF can affect water transport and uptake, carbon allocation, and SCP differently depending on plant genotype. Consistent with our first hypothesis, *Geopora* colonization improved water uptake in the drought tolerant genotype, but contrary to our hypothesis, the effect of the same EMF on water uptake of drought intolerant seedlings was the opposite. Plant genotype is known to influence EMF colonization^38^, community structure^39,40^, and EMF enzyme activity^41^. Ecological studies of drought in forests often emphasize differences in species composition of both trees and EMF. Our results suggest that, in addition to species, plant genotype is of critical importance in determining the effects of plant-fungal interactions on plant water availability and use, which can have cascading impacts on ecosystem properties such as carbon turnover, and water and nutrient utilization^42^.

Although the differences in the effects of a *Geopora*-dominated EMF rhizosphere community on the drought tolerant and intolerant pinyons were more extreme and complex than we hypothesized, they are consistent with *Geopora* colonization improving performance of the drought tolerant compared to drought intolerant plants observed in dry conditions in the greenhouse and the field^31,32^. *Geopora* colonization improved water uptake and transport more in the drought tolerant than drought intolerant genotype as hypothesized. The detrimental effect of the EMF colonization on water transport and uptake in the drought intolerant genotype was unexpected but could explain the consistently lower abundance of *Geopora* in drought intolerant pinyons^35^.

Previous studies show that EMF colonization of plant roots can lead to improved water availability^7^. They also suggest that this could result in faster growth with increased cell size^29^ and associated increase in xylem vulnerability to embolism, necessitating a more positive SCP^12,27,28,43,44^. Consistent with our second hypothesis, the inoculation treatment influenced plant growth more in the drought tolerant than in the drought intolerant seedlings, but contrary to our hypothesis and previous studies^31,32^, we observed a decrease in aboveground growth of the drought tolerant genotype when inoculated with live *Geopora*. This inconsistency may be explained by differences in plant age or water availability. Our seedlings were well-watered and younger than the plants exposed to drought in previous studies^31,32^. We also selected seedlings of similar above-ground size for water transport rate measurements to exclude the effect of plant size.

The reduction in aboveground growth with inoculation in drought tolerant seedlings could also be explained by differential belowground allocation between the two tree types. Drought tolerant seedlings receiving live *Geopora* maintained similar investment in root biomass and root length as their sterile inoculated counterparts, while also investing heavily in EMF. By contrast root length was > 50% lower in drought intolerant seedlings when inoculated with EMF. Ectomycorrhizal fungi are energetically costly; a recent meta-analysis calculated that host plants allocated an average of 13% of their net primary production to the external mycelium of EMF^45^. This energetic cost estimate is conservative as it does not include the cost of internal hyphae, exudates or sporocarps^45^. The large belowground investment by drought tolerant seedlings and adult trees appears to facilitate aboveground growth during drought^31,32^ but may come at the cost of aboveground growth in well-watered conditions. Reduced aboveground growth relative to nonmycorrhizal plants has been observed in several species of tree seedlings during early life as EMF establish mycelium in the soil^46–48^. However, reduced aboveground growth also promotes plant drought tolerance by inducing smaller and less vulnerable xylem conduits and a more favorable root:shoot ratio. Therefore, the carbon cost of maintaining EMF, while a disadvantage in competition for aboveground growth during well-watered conditions, might further promote plant drought resistance and survival under drought.

The opposing effects of *Geopora* inoculation on root length (Fig. 2) and root:shoot ratio (Fig. 3) highlight the contrast in traits related to drought tolerance and survival of these genotypes favorably balancing structures related to water supply and demand in the drought tolerant seedling^49^. The reduction in root length in live-inoculated drought intolerant seedlings is in line with previous findings showing that EMF use various signaling molecules to influence host root metabolism and morphology^50^, and plants that depend on EMF symbiosis develop coarser root systems with fewer root hairs than plants that rely less on symbiosis^51^. The reduced root length is explained by metabolic savings obtained by using the symbiont to support water and nutrient uptake^51^. In our experiment, the reduced root system of the drought intolerant seedlings was detrimental for water uptake and transport. This suggests that *Geopora* colonization failed to support water transport of the drought intolerant genotype despite reduced root growth. In contrast, in the drought tolerant genotype, inoculation both supported water transport and maintenance of high root biomass, which could be explained by improved water relations in the drought tolerant seedlings leading to increased carbon assimilation and allocation toward maintaining root growth and supporting the beneficial EMF, rather than promoting aboveground growth. While relatively costly during well-watered conditions, symbiotic rhizosphere fungi are a beneficial carbon investment promoting plant growth and survival under drought, and maximizing soil water access^31,52^.

In line with the reduced aboveground growth, the live-inoculated drought tolerant seedlings showed slightly lower SCP than the live-inoculated drought intolerant seedlings (Fig. 4a), but SCP seemed to be more closely related to the genotype than inoculation treatment or plant size. Lower SCP might be beneficial to promote survival under drought by allowing photosynthesis to continue under drier conditions^14^. The slight difference in SCP is thus in line with better drought survival of the drought tolerant genotype. More interestingly, however, our results suggest that SCP can be affected by EMF root colonization via its impacts on water availability (Fig. 4b), indicating that SCP may not be completely intrinsic to plant species, genotype and anatomy as previously thought. The SCP range observed in the drought tolerant genotype was large enough (−3.5 to −1.5 MPa) to warrant differential (an)isohydry, or desiccation tolerance/avoidance, categorization^18,28^. Supporting our hypothesis that EMF colonization would increase SCP, we observed a significant positive correlation between root EMF colonization and SCP in the drought intolerant genotype (Fig. 4b). But, contrary to our expectation, this correlation was not related to increased water uptake and consequent effects on plant growth. Instead, it was associated with reduced water uptake and root length. In the drought tolerant genotype, SCP decreased with increasing EMF colonization, but the relationship was not significant. Together with the opposing trends in root:shoot ratio with increasing EMF colonization (Fig. 3c), these results suggest that SCP in pinyon pine might decline with increasing water availability.

This finding, while contrary to expectations based on plant hydraulic theory, supports recent modeling studies suggesting that SCP is affected by soil-root hydraulics^22,53^, and is in line with the importance of water uptake and transport for plant performance and survival under drought. A SCP that declines with increasing water availability can explain the better growth of the *Geopora*-colonized drought tolerant seedlings under drought conditions^31,32^. Lower SCP would allow continued photosynthesis and growth under drier conditions. This finding also brings us closer to understanding stomatal control under drought. There is evidence that isohydric plants, like pinyon pine, might rely on hormonal signals (e.g., ABA) to trigger stomatal closure under drought, while anisohydric plants rely more on pressure signals^54^, but see also^55^. Previous observations of higher water availability leading to higher SCP are consistent with the pressure signal control, but do not consider the potential for microbial symbionts to significantly alter water availability and hydraulic resistance, as well as produce phytohormones like ABA^56^ that could influence stomatal control.

Our results also suggest that the effects of EMF on plant hydraulics manifested at the soil-root interface and EMF influence on plant growth rather than through enhanced water scavenging by fungal mycelia^7,10^. Live inoculation did not change root length or biomass in the drought tolerant seedlings compared to sterile inoculation, and the increase in water uptake and water flow velocity in the roots was not accompanied by a change in soil water flow rate. These results suggest that there was no change in water uptake area in the root structures of the live- and sterile-inoculated drought tolerant seedlings, and fungal mycelia did not significantly affect water movement outside the roots. This is consistent with the structure and growth form of EMF in the genus *Geopora* which form short distance hyphal exploration types^32,57^. The lack of long distance exploration hyphae could explain why root EMF colonization in the drought intolerant genotype was not sufficient to compensate for the loss of root length. However, our results from the drought tolerant genotype suggest that fungi with short-distance hyphal exploration types can play a critical role in water uptake and transport when compatible with the plant. This may occur through altered root anatomy, including the root hydrophilic properties that influence water uptake through the apoplastic pathway^10^, or production of aquaporins, major intrinsic proteins that can facilitate water movement into plant roots through the symplastic pathway^10,58^.

All in all, our results show that EMF colonization can differently affect plant hydraulics, carbon allocation, and desiccation tolerance under drought in different plant genotypes. These differences might lead to the divergent genotype preferences for EMF within the same plant species, as well as to differential genotype survival under drought. Our results also show that microorganisms can have significant effects on both plant structure and function, including important functional traits, like SCP, previously thought to be intrinsic to species. This finding opens new possibilities for using microorganisms to improve plant drought tolerance by directly targeting functional traits, rather than focusing only on growth and root structure, even if, for some plant species, genotype-level microorganism specificity is warranted.

## Methods

### Plant growth and inoculation

Pinyon pine seedlings were grown from two different maternal seed sources. Seeds from both drought tolerant and drought intolerant trees were obtained from a long-term study site near Sunset Crater National Monument, Arizona, USA^31^. Seeds of similar mass (0.30–0.35 g) were grown in 75% sand, 25% Los Alamos NM natural soil sieved with #200 mesh (0.074 mm grid size). The soil type of the natural soil was sandy loam with roughly 60% sand, 20% clay and 20% silt^59^. We used this soil mixture because it facilitated neutron imaging and was similar in texture to the low nutrient soils used for our previous studies of *P. edulis* response to *Geopora*^31^. Prior to planting, the field soil and sand mix was homogenized by thorough mixing, and sterilized by moistening the soil, placing it in covered dishes and microwaving on high power for seven minutes two times^60^. This method was used effectively to sterilize soil in previous experiments on *P. edulis*^31^. No fertilizer was added to the soil. To allow for water uptake imaging, soil was placed in custom-made rectangular aluminum containers (size 15 cm × 20 cm × 2.5 cm W × H × D) with an aluminium mesh bottom to allow sufficient drainage. It was necessary to use aluminum containers because neutrons, used for imaging water movement in the roots, cannot penetrate the plastic pots typically used in greenhouse studies. Half of the seeds from each mother source were inoculated with 5 g of *P. edulis* roots and adhering soil collected from drought tolerant maternal trees growing near Sunset Crater AZ (live inoculated treatment), and half were inoculated with similar but microwave-sterilized material (sterile-inoculated treatment) forming a crossed experimental design. We used inoculum only from drought tolerant trees as they are dominated by members of the genus *Geopora*, which contributes to the greater growth in drought conditions of drought tolerant *P. edulis*^31^. Using a *Geopora* dominated EMF community allowed us to determine if this fungal genus contributes to drought tolerance by improving water uptake in *P. edulis*. The seeds were planted on Dec 11, 2015, and seedlings were inoculated on January 4, 2016. Seedlings were grown in the Northern Arizona University research greenhouse under 12:12 supplemental light with temperatures of 24–27 °C during the day and 13–16 °C at night. The seedlings were watered to field capacity every 3 days and moved to Los Alamos National Laboratory for imaging on Jan 17–19, 2017.

### Neutron imaging

Water uptake of the seedlings was measured using neutron radiography imaging with heavy water (D_2_O) as contrast agent using a particle accelerator flight path P05 of the Lujan Center at Los Alamos National Laboratory^61^. For these measurements, we were granted 72 h of measurement time at the accelerator via a user application. Because water flow in coniferous trees is slow (~ 2 to 5 cm/h)^62^, and the transport distances in our setup needed for water movement detection through the plant were ~ 25 cm, the number of plant replicates per treatment measurable in the allocated time was limited. To ensure an experimental design with balanced sample size in each treatment, the water uptake measurements were made on six seedling pairs of live and sterile inoculation treatments, three pairs from drought tolerant seed sources and three from drought intolerant seed sources (n = 3 per treatment). The twelve seedlings for imaging were selected from a pool of 16 based on their size and photosynthesis rates to form as equal pairs as possible and eliminate any obvious differences in water transport capacity between groups. Therefore, prior to forming the pairs and imaging, maximum photosynthesis, and stomatal conductance rates of all the seedlings were measured using an infrared gas analyzer (Licor 6400, Licor Inc.). The gas analyzer was set at 1500 μmol m^−2^ s^−1^ PAR (above photosynthetic saturation for pinyon pine determined by measurements in field conditions for other studies (see e.g.^63^), 200 ml/s flow rate, 400 ppm CO_2_ concentration, and relative humidity at ~ 10% to mimic natural conditions in the middle of the day. Once the absence of differences between the groups was confirmed (Fig. S1), the seedlings were allowed to dry for five days to ensure ample water uptake during imaging. The 12 seedlings selected for neutron radiography were also used for all other measurements for consistency.

For imaging, the containers of the selected plant pairs were taped together with aluminum tape so that both plants were visible in the neutron beam (Fig. S2) and could be watered using a common plastic tray and measured simultaneously to ensure equal conditions for robust comparison of the effects of live and sterile inoculation on water uptake rates. To allow application of the D_2_O tracer without interruption to imaging, a PVC tube reaching outside the imaging chamber was attached to the watering tray to allow adding the tracer at a desired moment. A 12 W LED growth lamp (AgroLED, Spain) producing 550 μmol m^−2^ s^−1^ PAR was placed above the plants to induce stomatal opening. To estimate transpiration rates, the leaf temperature of each plant was measured with a small copper-constantan thermocouple attached on one of the needles. Leaf temperature was then compared to temperature measurements next to the plants with a similar thermocouple in the same environment. Temperature data was collected every 1 min using a Campbell CR1000 data logger (Campbell Scientific, Logan, UT, USA), and transpiration rates were calculated from the temperature difference using the leaf temperature model^64^.

For each plant pair, neutron radiography images were taken in pulses of 19 images over the course of approximately 110 s. To form a time series of images, these pulses were repeated continuously. The seedlings were initially imaged for approximately 10 min. After the initial 10 min, 100 ml of heavy water (D_2_O) was added to the plastic tray using a syringe and tubing. The uptake of D_2_O was imaged for 400–800 min (~ 7 to 13 h) until D_2_O reaching the plant stem and needles was confirmed by a clear neutron intensity increase at these tissues.

To compensate for the low number of replicates and confirm the repeatability of our results, each plant pair was imaged a second time, now using H_2_O as the contrast agent replacing D_2_O from the previous imaging in plant tissues. For this, the plants were removed from the neutron beam and allowed to dry for 8–12 h with no additional water to ensure ample water uptake. Once ready, they were again placed to the neutron beam, imaged or 10 min before applying 100 ml of H_2_O via the tube and watering tray and imaged continuously until H_2_O reaching the plant stem and needles was confirmed by a clear neutron intensity drop at these tissues.

After imaging, the plants were kept in quarantine for a period of ~ 8 days for any residual radioactivity to cool off. During this period the plants were kept under the growth lamp with a day/night cycle of 14/10 h and watered to field capacity every 3 days.

### Plant harvest

Once released from quarantine, the seedlings were moved to the laboratory where the fresh above and below ground biomasses, and SCP were measured. The aboveground biomass was collected, and the entire shoot used to determine SCP with simultaneous leaf water potential (pressure chamber method (pressure chamber Model 1005, PMS Instruments Company, Albany, OR, USA) and stomatal conductance measurements (Licor 6400; with similar settings as prior to imaging) using the bench drying method^65^. SCP was determined by fitting a Weibull probability distribution function (similar to Eq. (1) below) to the stomatal conductance data as a function of leaf water potential (Fig. S3). SCP was taken as the leaf water potential at 12% of maximum stomatal conductance^34^. Projected leaf areas of the seedlings were measured by scanning the needles and analyzing the scanned images with ImageJ software. The roots were separated from the soil using a gentle spray of water, weighed, wrapped in moist paper towels, placed in sealable plastic bags, and shipped overnight to Northern Arizona University for ectomycorrhizal fungal and root structure analysis. Finally, the stems and needles were dried in a drying oven at 65 °C for 48 h and weighed for dry biomass, and the same measurement was conducted on the roots after the fungal and root structural analyses.

### Root and EMF analyses

For root structural analysis, the entire root system of each seedling was scanned using the WinRhizo system (Regent’s Instruments, Inc., Canada) to obtain total root length. Root colonization by EMF was measured on each seedling by counting the number of living ectomycorrhizal root tips relative to non-colonized root tips based on differences in their morphology^66^. All living ectomycorrhizal root tips from each seedling were also characterized morphologically based on color, texture, hyphal quantity, and structure^67^. DNA was extracted from three to five root tips per morphotype per seedling, using the High Molecular Weight DNA Extraction protocol^68^. Polymerase chain reaction (PCR) was performed to amplify the internal transcribed spacer (ITS) region of the rRNA of the fungal genome with the ITS1-F (CTTGGTCATTTAGAGGAAGTAA) and ITS4 (TCCTCCGCTTATTGATATGC) primer pair^69,70^, using KAPA Taq Hotstart (Kapa Biosystems, Wilmington, MA, United States). Successfully amplified PCR product was purified and then cycle sequenced using BigDye Terminator Mix 3.1 (Thermo Fisher Scientific Inc.). Sequencing was performed on an ABI 3730xl Genetic Analyzer (Applied Biosystems, Foster City, California, United States) at Northern Arizona University. When amplification or sequencing of a morphotype was unsuccessful, an additional root tip from that morphotype from that tree was processed. We used BLASTn^71^ to query sequences against the NCBI DNA sequence database (https://www.ncbi.nlm.nih.gov/genbank/) using 98% similarity as a cutoff for species identification.

### Calculations of water uptake

The water uptake rates of both roots and soil were calculated from the neutron radiography images. First, each pulse of 19 images was averaged to produce a single image every 110 s. These average images were then stacked on top of each other to create a stack the length of the image run. The stack of images was then cleaned of outlier images using the *Plot Z-axis Profile* and *Delete Slice* tools in ImageJ, and then subjected to standard open and closed beam correction to remove the effects of changing background and beam intensity. The soil layer in each image throughout the stack was divided into four equal segments, each 200 pixels in height (Fig. S2). In each 200 pixel segment, both a root and a soil time series of neutron intensity was produced. To create the root time series, a polygon roughly 3000 square pixels in size, running from the top of the 200 pixel segment to the bottom, was selected, following the curvature of the root. To create the soil time series, a 40 × 200 pixel rectangular polygon was selected immediately to the left and right of the root polygon. Once these polygons were selected, the *Plot Z-axis Profile* tool of ImageJ was used to calculate the average intensity within each polygon for each image of the stack. To separate the root from the soil in front of and behind the root, the average intensity of the root polygon was divided by the average intensities of the two soil polygons next to the root segment. The average intensities from each root and soil polygon in each image of the stack were then plotted over time.

In the root time series stacks, D_2_O uptake can be detected as a sudden increase in intensity because the neutron absorbance of D_2_O is lower than that of H_2_O^72^. Similarly, in the replicates where H_2_O was used, there was a sudden decline in intensity. The inflection point can be interpreted as the moment when D_2_O (or H_2_O) has reached the measurement height, and the steepness of the curve and time to saturation reflect hydraulic properties of the material^73^. To determine the inflection points, a Weibull probability distribution function was fit to the time series plots:

1 $$I=1-(\frac{1}{1+{e}^{\left(a\left(t-b\right)\right)}})$$

where *I* is the signal intensity normalized to vary between 0 and 1, *t* is time in minutes since the beginning of the experiments, and *a* and *b* are the Weibull fit parameters determined by fitting this function to the intensity data using the least squares method. The point in time at which D_2_O (or H_2_O) uptake begins (*T*_*uptake*_; see Fig. S2) was then determined as the point where the tangent to the inflection point of the Weibull function crosses the x-axis, calculated as

2 $${T}_{uptake}=\frac{2}{a}+b$$

The points where the tangent to the Weibull function crossed the x-axis at each height, measured in minutes from the beginning of the image run, were then plotted as a function of the distance from the bottom of the image (D_2_O or H_2_O source). The flow velocity was calculated as the slope of a linear model fit to the data (distance/*T*_*uptake*_, m s^−1^). The flow velocities were converted to plant water uptake rates (m^3^ s^−1^) by multiplying the velocities with stem surface area at the root-to-stem transition point that was calculated from xylem diameters measured from neutron images at the soil surface assuming a cylindrical stem. This approximation for water uptake rate is based on mass balance and the plant pipe model theory according to which all the water collected by the roots has to pass through the stem below any foliage (see e.g.^74^). While the measured flow velocity in the roots is the most direct measure of water movement rate in the plant, the plant water uptake rate, calculated this way, normalizes the values for plant size and eliminates the effects of slight differences in seedling size on the results. The water flow velocity in the soil was calculated similarly to water flow rate in roots.

### Statistical analyses

The influence of inoculation and plant maternal seed source on water flow in roots, plant water uptake, plant transpiration, water flow in the soil, root colonization by EMF, root length, root biomass, aboveground biomass, leaf area, and plant SCP was analyzed using a two-way ANOVA with Tukey’s post hoc test with seedling type and inoculation as fixed effects. This analysis was conducted for treatment averages (EMF colonization, root length, root- and aboveground biomass, leaf area, and SCP). To ensure a robust comparison between live and sterile inoculation treatments for water flow rates in roots and soil, plant water uptake and transpiration rate, we calculated the effect of inoculation on these metrics by comparing the plants in each pair measured simultaneously and present the average change in water flow and uptake due to inoculation (i.e., difference in water flow and uptake between live and sterile inoculated plants; positive change means an increase due to inoculation). Analysis of the differences in the average change was determined similarly to the difference between treatment averages for the other metrics. Here we pooled the repeated measurement to one dataset. While the effects of inoculation on water transport in roots and soils was not always exactly identical in value, the difference between repeated measurements was less than 10% and the direction of the impact was maintained, and therefore pooling was justified. To examine the relationships between EMF colonization of roots, and plant structural and functional traits, we used linear regression analysis, selecting the model that minimized least squares, and evaluated the significance of the model predictive value using F-statistics. The calculations were conducted with Matlab version 2019a Statistical toolbox (Mathworks.com).

### Plant material permissions, licenses, and voucher specimens

*Pinus edulis* seeds and soil for inoculum were collected from long-term study trees with permission from the United States Forest Service. We implemented experimental research on *P. edulis* according to institutional, local and national guidelines. We do not have voucher specimens for deposit as the seedlings were destructively harvested at the end of the experiment as described in the methods section of the manuscript. The seedings are from a common tree species in the southwestern United States, *Pinus edulis* Engelm. which was formally described by George Engelmann in 1848.

### Supplementary Information

Supplementary Information.

## Data Availability

The datasets used and/or analyzed during the current study are available from the corresponding author on reasonable request.
