# Supplementary material for: Benefits of symbiotic ectomycorrhizal fungi to plant water relations depend on plant genotype in pinyon pine
Source: Sci Rep. 2023 Sep 2;13:14424. doi: 10.1038/s41598-023-41191-5 (PMC10475095; doi:10.1038/s41598-023-41191-5)
Supplement: Supplementary file 1 — Supplementary Information. [file 41598_2023_41191_MOESM1_ESM.docx]

**Supplementary materials**

Benefits of symbiotic ectomycorrhizal fungi to plant water relations depend on plant genotype in pinyon pine

Sanna Sevanto, Catherine A. Gehring, Max G. Ryan, Adair Patterson, Adrian S. Losko, Sven C. Vogel, Kelsey R. Carter, L. Turin Dickman, Michelle A. Espy, Cheryl R. Kuske


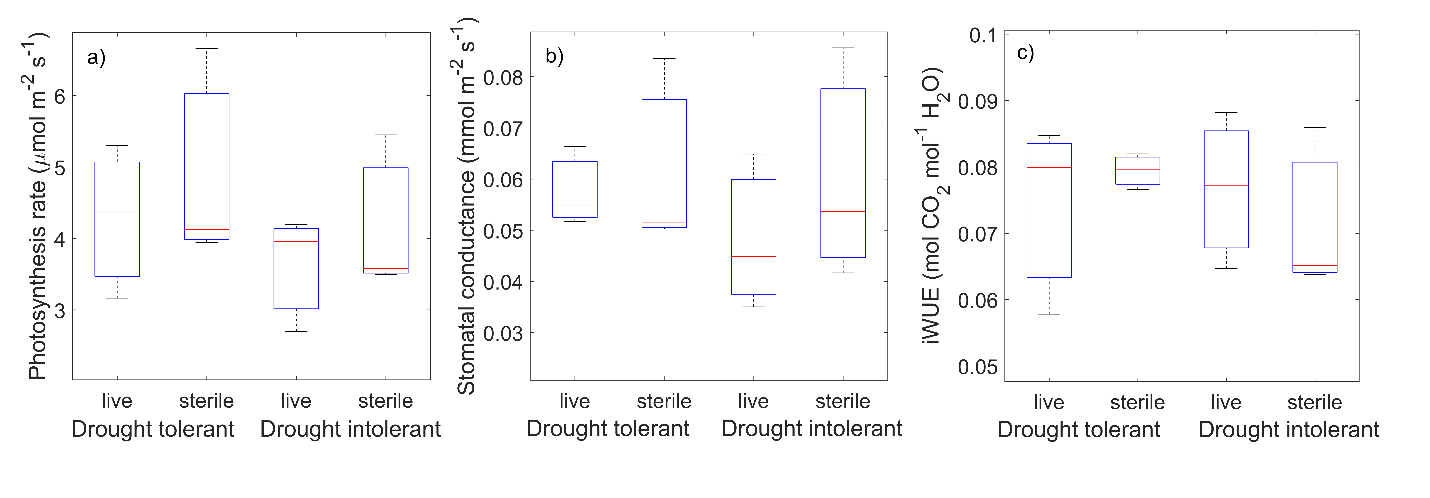


**Figure S1:** Effect of live and sterile inoculation treatments on photosynthesis (a), stomatal conductance (b) and intrinsic water use efficiency (iWUE; the ratio of photosynthesis to stomatal conductance) (c) of the drought tolerant and intolerant pine seedlings. There were no differences between the treatments (multi-way ANOVA, p>0.05). The data presented here was collected prior to neutron imaging. The leaf area for each measurement was estimated from photographs taken after the measurement because harvesting the leaves at this time point was not possible.


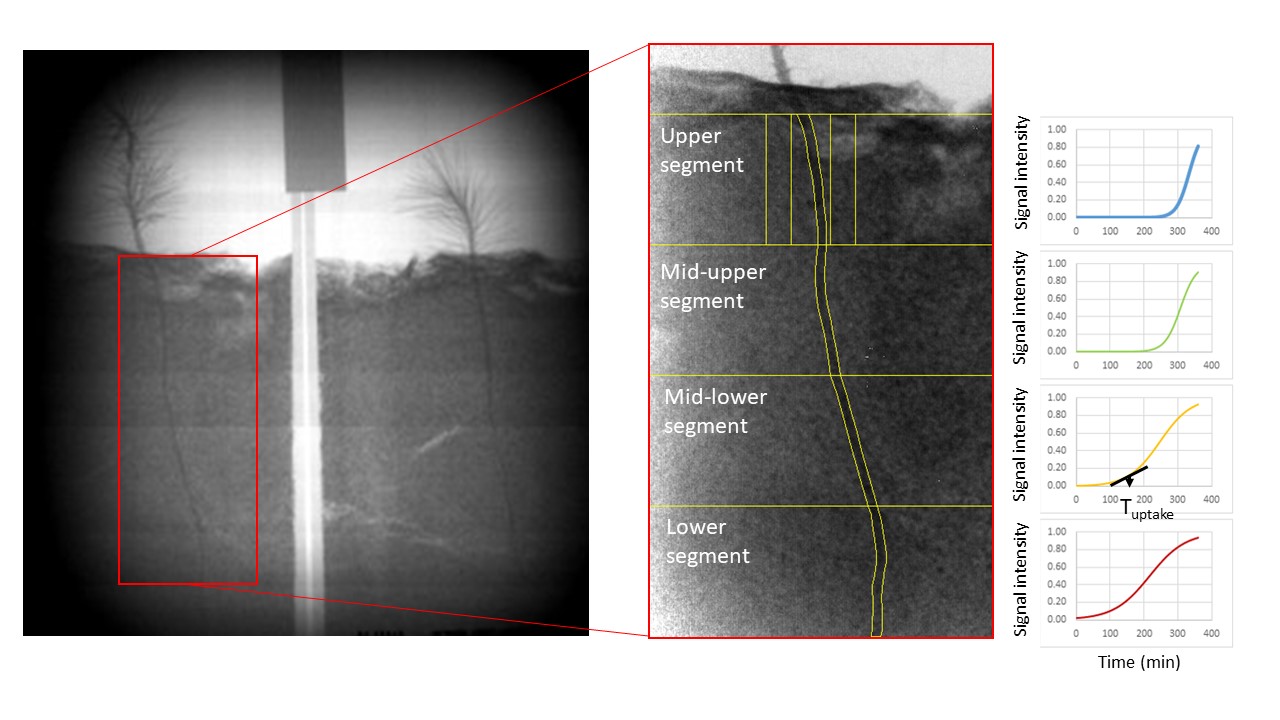


**Figure S2.** An example of how the water flow velocity and uptake rate were obtained from neutron radiography images. Pairs of live- and sterile inoculated pinyon pine seedlings were imaged with neutron radiography (left). To calculate the root water uptake rate, the main root of each plant was divided into four equal-size segments (middle), and a Weibull equation was fitted to the signal intensity changes at these segments (right; the different colors in the intensity graphs are used to distinguish between the segments). Water uptake rate was calculated from the slope of a linear fit to the points at which the D_2_O signal became visible at each height determined using the first inflection point of the curves (*T_uptake_* marked for example only in one of the intensity graphs). Effects of the soil were removed by selecting equal-size soil segments on both sides of the root and subtracting their average from the signal intensity at the root marked as the top segment. This protocol was repeated for each segment and both for both live and sterile inoculated plants to allow comparison of water uptake rates. Water flow in the soil was calculated with a similar protocol but using segments outside of the root zone.


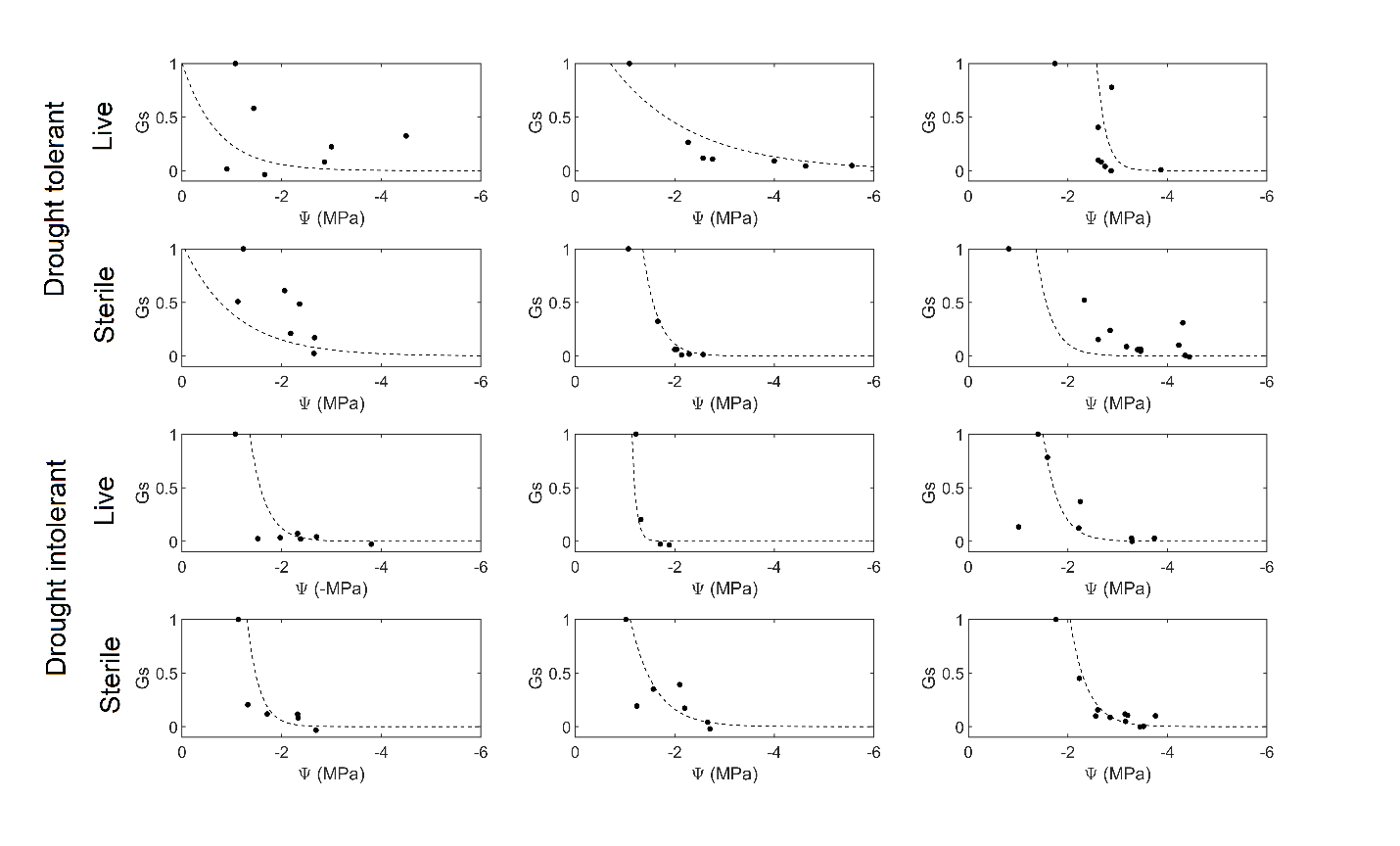


**Figure S3:** The data used for determining stomatal closure point (SCP) for the live- and sterile-inoculated drought tolerant (two top rows) and drought intolerant (two bottom rows) seedings. Stomatal conductance (Gs) on the y-axis is normalized to its maximum as is customary when determining SCP (Skelton et al. 2015). The dashed line shows the fitted Weibull equation (eq. (1) in the manuscript) for each case. Stomatal closure point is the leaf water potential (Ψ on the x-axis) representing the inflection point of the fitted curve.

**References**

Skelton, R. P., West, A. G. & Dawson, T. E. Predicting plant vulnerability to drought in biodiverse regions using functional traits. *Proc. Natl. Acad. Sci. U.S.A* **112,** 5744-5749 (2015).
